# Effect of addition of processed bambara nut on the functional and sensory acceptability of millet‐based infant formula

**DOI:** 10.1002/fsn3.618

**Published:** 2018-03-09

**Authors:** Samaila James, Ngozi Iwanger Akosu, Yakubu Caleb Maina, Amina Ibrahim Baba, Lilian Nwokocha, Suleiman James Amuga, Yohanna Audu, Maxwell Yemmy Mitchel Omeiza

**Affiliations:** ^1^ Department of Food Science and Technology Federal University of Technology Minna Nigeria; ^2^ Department of Hospitality and Tourism Management Delta State Polytechnic Ogwashi‐Ukwu Delta State Nigeria; ^3^ Federal University Kashere Kashere Gombe State Nigeria

**Keywords:** bambara nut, fermentation, infant, millet, sprouting

## Abstract

Fermentation and sprouting have been shown to increase nutrient bioavailability and modify the functional properties of foods. Application of these methods in the preparation of infant foods and complementing cereals with legumes will address nutrient density and viscosity problems associated with infant foods. Infant foods were formulated from blends of treated bambara nut and pearl millet. Functional properties, pasting properties, and sensory acceptability of the blends were studied. Millet and bambara nut were soaked separately in water and allowed to ferment for 48 hr at room temperature. While for sprouting, millet and bambara nut were soaked for 12 and 24 hr, respectively, at room temperature, and soaked seeds were separately sprouted for 48 hr. After fermentation and sprouting, the seeds were oven‐dried and then milled into a flour of 0.6 mm size. The flours were formulated to six (A, B, C, D, E, and F) complementary diets. The results show that sample E had the suitable water absorption capacity, while, for oil absorption capacity, various blends showed suitability except samples A and B. In terms of swelling capacity, sample F (6.52 ± 0.01%) was the suitable at 60°C. Treatment and blending significantly influenced trough, final viscosity, and pasting time. In sensory acceptability, sample B was adjudged the best. This study revealed that sample C was the best in respect to functional and pasting properties for infant food; however, sample B received the best general acceptability.

## 1 INTRODUCTION

Complementary foods are foods other than breast milk introduced to infants to raise their nutrients supply. From 6 months onward, breastfeeding alone does not adequately supply the nutrient requirement of an infant (Dewey & Brown, 2003). At this period of development, semisolid food gruels are introduced to infant and this serves as a way of introducing the infant to the family food According to the World Health Organization (WHO), complementary feeding should be timely, adequate, appropriate, and given in sufficient quantity (WHO, 2003). Adequate nutrition during infancy and early childhood is critical to the development of the child's full potential (WHO, 2002). The period of 6 to 18 months of complementary feeding is characterized by physiological, immunological, mental, and rapid physical growth (WHO, 1998). It has been reported that proper brain development occurs in the first 2 years after birth (Evidence Review Series, 2008; WHO, 1998). However, this transition period makes infants vulnerable to so many diseases and infection (UNICEF, 1998).

Most local complementary foods served to growing infants are inadequate in protein, energy, essential amino acids, and micronutrients (Nwosu, Nnam, Ibeziako, & Maduforo, 2014; WHO, 2003). They are often high in dietary fiber and antinutrients thereby making nutrient utilization in infants difficult and leading to serious nutrient‐related health problem and high incidence of malnutrition (Nnam, 2000). Asindi, Ibia, and Udo (1990) reported that 30%–40% deaths of preschool children in Nigeria are associated with malnutrition. National Population Commission (National Nutrition and Health Survey (NNHS) [Nigeria], 2015; National Population Commission (NPC) [Nigeria] and ICF International, 2014) revealed that more than 40% of under‐five age children are stunted in growth, 90% are wasting, 25% underweight, and 60% die to malnutrition‐related causes. Furthermore, only 15%–17% nursing mothers exclusively breastfeed their babies (National Nutrition and Health Survey (NNHS) [Nigeria], 2015). This report implies that infants are introduced early to complementary foods even though their digestive system is still tender and immature and hence not ready for such foods. The resultant effects would be the development of early digestive problems, adverse physiological, and immunological responses (Kathryn & Seth, 2012). Several strategies have been employed such as nutrition education of mothers on healthy infant and young child feeding practices, production of nutrient‐dense complementary foods, and micromineral fortification among others (GAIN, 2013; Kathryn & Seth, 2012).

In developing countries such as Nigeria, complementary foods are majorly made from cereals such as millets, corn, sorghum, rice, and starchy tubers such as cocoyam, sweet potato among others. Their mode of preparations has not been optimized to provide the required nutrients. Hence, consumption of these starchy gruels which are inadequate in protein, energy, essential amino acids, and micronutrients has been the major cause of nutrient‐related illnesses, weak immunological response, and retarded body growth in infants (National Nutrition and Health Survey (NNHS) [Nigeria], 2015). Situation Standing Committee on Nutrition (World Nutrition Situation Standing Committee on Nutrition (SCN), 2004) reported that protein‐energy malnutrition and micronutrients deficiency among infants, children, and pregnant women account for 50% childhood morbidity and mortality in the developing world.

Nutritionists and food scientists at individual and organizational levels have been working toward the provision of nutritious, cheap, and readily available dietary supplements for young children in the developing world (UNICEF, 2001). Ingredients used for such formulations are derived from dietary staples available and affordable in the region of interest. For example, Global Alliance for Improved Nutrition (GAIN) (GAIN, 2013) has been supporting companies and agencies in the development, production, and marketing of fortified complementary foods (FCF), complementary food supplements (CFS), and multiple micronutrients powders (MNP) to improve nutritional status of older infants and young children in several developing countries. A more effective, sustainable, and comprehensive food‐based approach has been advocated. Some of the problems associated with complementary feeding regime include safe preparation and storage, frequency of intake, nutrient density, viscosity problems, and age of introduction among others (Kathryn & Seth, 2012). Many local brands of weaning foods have been developed and adopted in most developing countries, Nigeria inclusive. A number of homemade complementary diets from blends of cereal, legumes, animal products, and fruits and vegetables have been reported (Adepeju, Gbadamosi, Omobuwajo, & Abiodun, 2014; Akinola, Opreh, & Hammed, 2014; Gazhim, Bintu, Modu, Falmata, & Zainab, 2015; Nnam, 2000; Nwosu et al., 2014; Odom, Udensi, & Iwe, 2013; Olapade & Aworh, 2012; Onoja, Akubor, Gernar, & Chinma, 2014; Steve & Olufunke, 2013). Most of the formulations were centered on only refining the cereal flour prior to blending. There is need to exploit some local processing methods such as sprouting and fermentation on local foodstuff that are shown to improve nutrients bioavailability, modify textural characteristics, predigest high molecular weight macromolecules, and reduce antinutrients contents.

Bambara nut (*Voandzeia subterranea* L. Thours) is a legume that is generally grown in west and central Africa (Christiana, 2009). Although bambara nut is grown extensively in Nigeria, it is one of the underutilized legumes in the country. It is a rich source of protein (20%–25%); its protein is reported to be high in essential amino acid methionine than other legumes and contains 6%–12% lipid (Stephens, 2011). The seeds are locally eaten in a number of ways. In eastern Nigeria, the seeds are milled into flour and used as a major ingredient in *Okpa* production; while in northern part, it is boiled and eaten with cereals grains or roasted and flavored with salt and eaten as a snack. In western part, the seeds are milled into flour and used for preparing *fufu*. In recent time, the seeds are used to fortify maize for pap (Okonkwo & Opara, 2010). However, the beany flavor, hard‐to‐cook property, and high antinutrients have contributed to its low applicability in food formulations.

Treating the seeds via sprouting and fermentation which modify the endosperm thereby generating free amino acids, vitamins, sugars, and certain minerals, as well as reducing the antinutrients, would be beneficial for inclusion in infant formulation to complement cereal flour. Blending treated bambara nut and millet flour will offer a complementary infant formula with optimal dietary requirement for infants. The process of treating the food ingredients by fermentation will improve their digestibility, nutritional value, texture, flavor, and taste, while germination will increase dietary fiber, B‐complex vitamins, proteins, and antioxidants and also eliminate/reduce harmful compounds such oxalate. Blending millet and treated bambara nut flours at the ratios used in this study provides the recommended protein requirements (16%) for infants. This was achieved via material balance. Therefore, the study examined the effect of sprouting, fermentation, and blending ratios on the functional properties and sensory acceptability of the formulated infant diet.

## 2 MATERIALS AND METHODS

### 2.1 Source of raw materials

Bambara nut and pearl millet were purchased from Kure Ultra‐Modern Market, Niger State, Nigeria.

### 2.2 Material preparation—fermentation millet and bambara nut

Bambara nut and millet were fermented as described by Chikwendu, Obiakor, and Maduforo (2014) with slight modification. The two raw materials were cleaned manually, washed in clean water after which they were soaked separately in cold water in a ratio of 1:3 weight by volume (w/v) and allowed to ferment for 48 hr at room temperature (28 ± 2°C). Fermented millet and bambara nut were thoroughly washed in clean water and oven‐dried separately at 60°C for 12 and 24 hr, respectively, and then hammer milled into a fine flour of 0.6 mm size. The flours were packaged differently in coded high‐density polythene bags for further analysis.

### 2.3 Preparation of sprouted millet and bambara nut

Method described by Okafor, Jane, Ani, and Gabriel (2014) was used to sprout millet and bambara nut with slight modifications. Millet and bambara nut were sorted, washed, and soaked in clean water for 12 and 24 hr, respectively, at room temperature (28 ± 2°C) with change in soaking water at 4‐hr interval to prevent fermentation. Soaked seeds were separately spread on jute bag and covered with the same and allowed to sprout for 48 hr with sprinkling of water at 3‐hr intervals. After sprouting period, the seeds were evenly spread on oven trays and dried at 60°C for 12 and 20 hr, respectively, and then hammer milled into a flour of 0.6 mm size and packaged differently in high‐density polyethylene for further analysis.

### 2.4 Product formulation

The samples for this study were formulated, thus 100% sprouted millet flour, 100% fermented millet flour, 95% sprouted millet flour and 5% sprouted bambara nut flour, 95% fermented millet flour and 5% fermented bambara nut flour, 95% sprouted millet flour and 5% fermented bambara nut flour, and 95% fermented millet flour and 5% sprouted bambara nut flour, representing samples A, B, C, D, E, and F, respectively.

### 2.5 Functional properties

#### 2.5.1 Determination of bulk density

Method described by Onwuka (2005) was adopted. A 10‐ml graduated measuring cylinder was weighed and filled with the sample, and the bottom of the cylinder was gently tapped on the laboratory bench several times until there was no further diminution of the sample level after filling to the 10 ml mark.

$$\mathrm{Bulk}\;\mathrm{density}\;\left(g/\mathrm{ml}\right)=\frac{\mathrm{Weight}\;\mathrm{of}\;\mathrm{sample}\;(g)}{\mathrm{Volume}\;\mathrm{of}\;\mathrm{sample}\;(\mathrm{ml})}$$

#### 2.5.2 Determination of water and oil absorption capacity

One (1) gram of the sample was weighed into a conical graduated centrifuge tube, a warring whirl mixer was used to mix the sample, and 10 ml of distilled water was added and thoroughly mixed for 30 s. The sample was allowed to stand for 30 min at room temperature and then centrifuged at 5000×*g* for 30 min. The volume of free water (supernatant) was read directly from the graduated centrifuge tube. The water absorbed (total minus free) was multiplied by the density of water (1 g/ml). The same procedure was used for oil absorption except that oil was used instead of water (Onwuka, 2005).

#### 2.5.3 Determination of the gelation concentration

Method described by Onwuka (2005) was used for the determination of gelation capacity. A sample suspension of 2%–20% (w/v) in 5 ml of distilled water was prepared in test tubes. The samples in the test tubes were heated for 1 hr in a boiling water bath followed by rapid cooling under running cold tap water. The test tubes were further cooled for 2 hr at 4°C. The least gelation concentration was determined as the concentration when the sample from the inverted test tube did not fall or slip.

#### 2.5.4 Determination of foam capacity (FC)

Two (2) gram of the flour sample was blended with 100 ml of distilled water in a warring blender, and the suspension was whipped at 1600 rpm for 5 min. The mixture was poured into a 250‐ml measuring cylinder, and the volume was recorded after 30 s. Foam capacity was expressed as percent increase in volume using the formula:

$$\begin{array}{cc} & \mathrm{FC}\;\left(\%\;\mathrm{volume}\;\mathrm{increase}\right)\;=\frac{\mathrm{volume}\;\mathrm{after}\;\mathrm{whipping}-\mathrm{volume}\;\mathrm{before}\;\mathrm{whipping}}{\mathrm{volume}\;\mathrm{before}\;\mathrm{whipping}}\times 100 \end{array}$$

#### 2.5.5 Determination of swelling capacity and solubility index

The swelling capacity and solubility index were determined as described by AOAC (2000). One gram of the sample was accurately weighed and transferred into a clean dried test tube and weighed (*W*_1_), and then it was dispersed into 30 cm^3^ distilled water using blender. The resultant slurry was heated at temperatures of 60, 70, 80, and 90°C, respectively, for 30 min in a regulated water bath. The mixture was then cooled to room temperature and centrifuged at 500 rpm for 15 min. 5 ml of the supernatant was withdrawn and dried to a constant weight at 110°C in an oven. The residue was then represented as the amount solubilized in water.

$$\begin{array}{cc} \mathrm{Solubility} & \;=\frac{\mathrm{Weight}\;\;\mathrm{after}\;\mathrm{drying}\;\;\mathrm{of}\;\;\mathrm{supernatant}}{\mathrm{Weight}\;\;\mathrm{after}\;\;\mathrm{drying}\;\;\mathrm{of}\;\;\mathrm{dried}\;\;\mathrm{sample}}\times 100 \end{array}$$

### 2.6 Pasting properties

Pasting characteristics were determined with a Rapid Visco Analyzer (RVA), New Port Scientific RVA Super 4 Machine with serial number 2112582‐S4A made in Australia. The pasting properties included pasting temperature, peak viscosity, peak time, hot and cold viscosity breakdown, set back, and final viscosity which were read from the pasting profile with the aid of thermocline for windows software connected to a computer. Three and a half (3.5) gram of the samples were weighed into a dried empty canister, and then 25 ml of water was dispensed into the canister containing the sample. Paddle was placed inside the canister; this was placed centrally onto the paddle coupling and then inserted into the RVA machine. The measurement cycle was initiated by pressing the motor tower of the instrument. The profile could be seen as it is running on the monitor of the computer connected to the instrument. The 13‐min profile was used, and the time–temperature regime was also used. It was expressed as RVU (Relative Visco Analyzer Unit).

### 2.7 Sensory evaluation

The sensory evaluation was carried out by 20 respondents which comprised of nursing mothers who were students of the Department of Food Science and Nutrition, Federal University of Technology Minna. The samples were ranked on a 9‐point hedonic scale with 1 representing dislike extremely and 9 like extremely. Gruel was prepared from each of the samples by boiling 10% (w/v) slurry for 10 min and served immediately after preparation. Individual samples were presented in a random pattern and judged based on aroma, texture, taste, appearance, and overall acceptability. A glass of clean water was given to the respondents to rinse their mouth in between each determination to avoid discrepancies in taste.

### 2.8 Statistical analysis

The data obtained were subjected to analysis of variance (ANOVA), and separation of the mean values was carried out using Duncan multiple range test at the 5% significance level.

## 3 DISCUSSION

### 3.1 The functional properties of the samples

Table 1 shows the functional properties of the treated flour blends. The result indicates that sprouting, fermentation, and blending ratios significantly (*p* < .05) influenced the functional properties of all the samples; however, gelation capacity and bulk density were not significantly affected (*p* > .05). Sample B (100% fermented millet) was significantly high in water absorption capacity (*p* < .05) followed by 100% sprouted (sample A). Blending, however, significantly reduced water absorption capacity with sample E (0.87 g/cm^3^) having the least value. Water absorption capacity is an index of water absorbed and retained, and the result obtained in this study (2.07–0.87 g/cm^3^) agrees with 1.03, 1.29, and 0.77 g/cm^3^ for sorghum, pearl millet, and maize flours, respectively (Sing, Yadav, & Sharma, 2012). Low water absorption capacity is desirable for making thinner gruels with high caloric density per unit volume. This increases absorption of nutrients by infants and reduction in microbial activities due to low water activity thereby extending the shelf life of the product (Gomez & Aguilera, 1983). Significantly, high water absorption capacity in 100% fermented millet could be attributed to high hydrolysis of macromolecules which exposed their hydrophilic domains thereby increasing their affinity for water.

**Table 1:** Functional properties of the samples

Parameter	A	B	C	D	E	F
WAC (g/cm^3^)	1.90^b^ ± 0.06	2.07^a^ ± 0.03	1.57^c^ ± 0.03	1.80^b^ ± 0.06	0.87^d^ ± 0.03	1.50^c^ ± 0.06
OAC (g/cm^3^)	1.97^a^ ± 0.03	1.90^a^ ± 0.06	1.70^b^ ± 0.06	1.60^b^ ± 0.06	1.67^b^ ± 0.03	1.67^b^ ± 0.03
Gelation (w/v)	6.00 ± 0.00	4.00 ± 0.00	4.00 ± 0.00	4.00 ± 0.00	6.00 ± 0.00	4.00 ± 0.00
FC (%)	3.96^d^ ± 0.02	5.87^c^ ± 0.01	11.76^a^ ± 0.01	9.68^b^ ± 0.07	11.79^a^ ± 0.01	9.68^b^ ± 0.07
Bulk density (g/cm^3^)	0.43 ± 0.01	0.38 ± 0.01	0.33 ± 0.02	0.38 ± 0.01	0.39 ± 0.03	0.34 ± 0.01

Mean ± *SD* of triple determinations. Values followed by different subscript on a row are significantly different from each other (*p* < .05). A = 100% sprouted millet; B = 100% fermented millet; C = 95% sprouted millet and 5% sprouted bambara nut; D = 95% fermented millet and 5% fermented bambara nut; E = 95% sprouted millet and 5% fermented bambara nut; F = 95% fermented millet and 5% sprouted bambara nut; WAC, water absorption capacity; OAC, oil absorption capacity; FC, foam capacity.

The result for oil absorption capacity shows no significant difference between the blended samples (*p* > .05); however, 100% sprouted and fermented samples had significantly high values. The result in this study (1.97–1.60 g/cm^3^) was low compared to the values obtained for sorghum (8.1 g/cm^3^), pearl millet (8.2 g/cm^3^), and maize (8.5 g/cm^3^) (Sing et al., 2012). The result, however, agrees with 1.93 g/cm^3^ for cowpea protein isolate (Appiah, Asibuo, & Kumah, 2011).

Gelation is the ability of starch to absorb moisture, swell, and lost its bifrengence when heat is applied. Infant formulas with high gelation require less heat during preparation (Usman, Bolade, & James, 2016). Sprouting, fermentation, and blending ratios did not significantly affect the samples (*p* > .05). Forming capacity is the ability of the formula to foam, when water and heat are applied, and is dependent on soluble proteins. Samples A and B were significantly low in FC, while various blends had significantly high values. The result recorded in this study (3.96%–11.76%) agrees with 4.00%–11.33% for germinated tiger nut varieties (Chinma, Abu, Matti, & Salami, 2013). High foam capacity in infant diet is not desirable; however, preparation at reduced pressure minimizes its formation.

Bulk density is a measure of the heaviness of the flour. In this study, the result obtained (0.43–0.33 g/cm^3^) was low compared to results reported for sorghum (0.61 g/cm^3^), pearl millet (0.59 g/cm^3^), and maize (0.60 g/cm^3^) (Sing et al., 2012) and that of protein concentrate from cowpea seeds (0.925–1.09 g/cm^3^) (James et al., 2016). The result, however, is similar to 0.44–0.41 g/cm^3^ for maize‐based infant blend (Helen & Ragina, 2013). Lower bulk density promotes digestibility of the formula, especially among children with immature digestive system (Onimawo & Egbekun, 1998). The low bulk density in this study could be attributed to the treatments (fermentation and sprouting) which have the potentials of modifying the endosperm.

The solubility of the blends at different temperatures was significantly affected by processing methods (Table 2). The solubility of a protein is an important functional property as protein needs to be soluble in order to be applicable in food systems. Other functional properties such as emulsification, foaming, and gelation depend on the solubility of protein (Henshaw & Sobowale, 1977). In this study, the solubility of the samples increased with increase in temperature. This can be attributed to the fact that solubility of protein is influenced by temperature, ionic strength, and pH (Khalid, Elhardallou, & Elkhalifa, 2012). Exclusively, sprouted millet had the highest solubility at 70, 80, and 90°C, while sample C had the lowest solubility. High solubility of the gruels most especially at relatively high temperatures aids the uniform mixing of the constituents, and this gives a smooth and consistent gruel which is desirable for infant.

**Table 2:** Solubility of the samples at different temperatures

Solubility at (°C)	A	B	C	D	E	F
60	1.04^c^ ± 0.01	1.62^b^ ± 0.19	1.60^b^ ± 0.00	2.35^a^ ± 0.00	1.17^c^ ± 0.01	1.63^b^ ± 0.01
70	6.30^a^ ± 0.01	2.63^b^ ± 0.01	1.81^f^ ± 0.01	2.40^c^ ± 0.02	2.13^d^ ± 0.02	1.91^e^ ± 0.01
80	7.36^a^ ± 0.01	3.83^b^ ± 0.01	1.88^f^ ± 0.01	2.88^c^ ± 0.01	2.15^d^ ± 0.01	2.05^e^ ± 0.01
90	8.68^a^ ± 0.01	4.47^b^ ± 0.67	2.03^c^ ± 0.01	3.78^b^ ± 0.01	2.31^c^ ± 0.01	2.18^c^ ± 0.01

Mean ± *SD* of triple determinations. Values followed by different subscript on a row are significantly different from each other (*p* < .05). A = 100% sprouted millet; B = 100% fermented millet; C = 95% sprouted millet and 5% sprouted bambara nut; D = 95% fermented millet and 5% fermented bambara nut; E = 95% sprouted millet and 5% fermented bambara nut; F = 95% fermented millet and 5% sprouted bambara nut.

Swelling capacity is used to determine the amount of water that the formula will absorb and the degree of swelling within a given time and temperature. In this study (Table 3), the swelling capacity increased with increase in temperature; this is in agreement with Claver, Zhang, Kexue, and Zhou (2010) who reported that temperature increase and vigorous starch vibration break intermolecular bonds, thereby allowing hydrogen bonding sites to accommodate more water molecules. Low swelling capacity is an advantage in complementary feeding as it increases the nutrient density of the food, and the child is able to consume more in order to meet the nutrient requirement. Sample D had the highest swelling capacity at 60, 70, 80, and 90°C, while sample C had the least value.

**Table 3:** Swelling capacity of the samples at different temperatures

Swelling capacity at (°C)	A	B	C	D	E	F
60	7.59^d^ ± 0.01	7.81^c^ ± 0.00	6.59^e^ ± 0.01	10.08^b^ ± 0.01	10.92^a^ ± 0.01	6.52^e^ ± 0.01
70	8.41^e^ ± 0.01	14.49^b^ ± 0.01	7.56^f^ ± 0.01	14.99^a^ ± 0.01	11.01^c^ ± 0.00	9.14^d^ ± 0.01
80	10.51^d^ ± 0.01	16.76^b^ ± 0.00	8.71^f^ ± 0.00	17.91^a^ ± 0.00	11.59^c^ ± 0.01	10.02^e^ ± 0.01
90	14.73^c^ ± 0.01	17.29^b^ ± 0.01	8.77^f^ ± 0.02	18.13^a^ ± 0.01	12.87^d^ ± 0.02	12.49^e^ ± 0.07

Mean ± *SD* of triple determinations. Values followed by different subscript on a row are significantly different from each other (*p* < .05). A = 100% sprouted millet; B = 100% fermented millet; C = 95% sprouted millet and 5% sprouted bambara nut; D = 95% fermented millet and 5% fermented bambara nut; E = 95% sprouted millet and 5% fermented bambara nut; F = 95% fermented millet and 5% sprouted bambara nut.

### 3.2 Pasting properties of sprouted and fermented millet and bambara nut flour blend

Pasting characteristics is one of the most important properties that determine quality and aesthetic considerations. In the food industries, such properties affect texture, digestibility, and the applicability of starch‐based food commodities (Adebowale, Adegoke, Sanni, Adegunwa, & Fetuga, 2011). When an aqueous suspension of starch is heated above a critical temperature, granules swell irreversibly and amylose leaches out into the aqueous phase resulting into increased viscosity pasting (Brabet et al., 1998). The result of the pasting properties in this study (Table 4) shows that sprouting and fermentation processes did not significantly affect peak viscosity, setback, and pasting temperature (*p* > .05); however, trough, final viscosity, and pasting time and pasting temperatures were significantly influenced (*p* < .05).

**Table 4:** Pasting properties of the samples

Pasting properties (RVU)	A	B	C	D	E	F
Peak viscosity	63.73 ± 0.28	64.95 ± 0.95	62.10 ± 0.01	63.06 ± 0.17	63.79 ± 0.56	64.10 ± 0.78
Trough	1.37^c^ ± 0.04	1.63^c^ ± 0.03	2.85^b^ ± 0.15	2.10^b^ ± 0.01	3.22^a^ ± 0.11	2.43^b^ ± 0.25
Breakdown	17.71 ± 0.07	16.93 ± 0.05	16.68 ± 0.12	16.83 ± 0.07	16.44 ± 8.45	16.67 ± 0.33
Final viscosity	8.35^a^ ± 0.05	8.50^a^ ± 0.05	7.56^b^ ± 0.23	7.79^b^ ± 0.01	7.49^b^ ± 0.09	7.88^b^ ± 0.10
Setback	1.55 ± 0.05	1.85 ± 0.06	1.69 ± 0.09	1.74 ± 0.04	1.72 ± 0.06	1.77 ± 0.01
Pasting time (min)	5.39^bc^ ± 0.41	5.39^c^ ± 0.01	6.30^ab^ ± 0.20	6.56^a^ ± 0.16	6.89^a^ ± 0.12	6.71^a^ ± 0.28
Pasting temperature (°C)	74.28 ± 0.28	73.68 ± 0.23	72.58 ± 0.21	73.83 ± 0.07	73.50 ± 0.50	74.15 ± 0.85

Mean ± *SD* of triple determinations. Values followed by different subscript on a row are significantly different from each other (*p* < .05). A = 100% sprouted millet; B = 100% fermented millet; C = 95% sprouted millet and 5% sprouted bambara nut; D = 95% fermented millet and 5% fermented bambara nut; E = 95% sprouted millet and 5% fermented bambara nut; F = 95% fermented millet and 5% sprouted bambara nut.

Peak viscosity indicates the water‐binding capacity of the starch. It is the point at which gelatinized starch reaches its maximum viscosity during heating in water (Shimelis, Meaza, & Rakishit, 2006). In this study, samples did not show any significant difference. This implies that different treatment given and the blending ratio did not influence the peak viscosity. High peak viscosity maybe suitable for products requiring high gel strength and elasticity. The result obtained in this study (62.10–64.95 RVU) is suitable for infants who require low viscosity gruel. Trough measures the ability of the paste to withstand breakdown during cooling. It is the minimum viscosity value in the constant temperature phase of the RVA pasting profile (Adebowale et al., 2011). Treated blends had the highest values, which implies that the blends have the ability to remain undisrupted when subjected to hold period of constant high temperature and mechanical stress by rapid and continuous mixing. Infant food is expected to remain uniform without separation of layers either hot or at room temperature.

Breakdown measures the degree of disintegration of granules or paste stability. Zaidul, Norulaini, Omar, Yamauchi, and Noda (2007) reported that, at breakdown, swollen granules disrupt further and amylose molecules leach into the solution. In this study, samples did not show any variability. This implies that blending ratio and treatment had no influence on breakdown values. The higher the breakdown value, the lower the ability of the samples to withstand heating and shear stress during cooking. The result obtained in this study (16.44–17.71 RVU) was low compared to 41.58 RVU and 56.50–107.75 RVU for *Brachysteria euryloma* flour and cassava starch isolate, respectively (Ikegwu, Nwobasi, Odoh, & Oledinma, 2009; Ikegwu, Okechukwu, & Ekumankana, 2010). Starches with high breakdown are likely to produce unsuitable paste (Singh, McCarthy, & Singh, 2006). As low breakdown values are associated with stable gruels, the low values reported in this study would be suitable for infant food formulation.

Final viscosity defines the quality of a particular flour resistance during stirring. It also indicates the ability of starch to form paste or gel after cooling. Less stable starch paste is commonly accompanied with high value of breakdown. Samples A and B showed significantly (*p* < .05) high values, while various blends showed significantly (*p* < .05) low values. High values of the two samples could be attributed to noninclusion of bambara nut flour in them. Low final viscosity implies that the infant diet will form a low viscous paste rather than a thick gel on cooking and cooling. Also, the gruel will be a high caloric density food per unit volume. Hence, low value reported in this study is desirable for infant food preparation.

Setback viscosity is the phase in the pasting curve after cooling the paste that shows the tendency of the starch to associate and retrograde (James & Nwabueze, 2014; Ogundele, Ojubanire, & Bamidele, 2015). High setback values have been associated with a cohesive paste, while a low setback is an indication that the paste is not cohesive. Therefore, the higher the setback viscosity, the lower the retrogradation during cooling and the lower the starting rate of the product made from the flour (Adebowale & Lawal, 2003). In this study, samples did not differ significantly in setback value; however, the values (1.55–1.85 RVU) obtained were low implying less susceptibility of the flour to retrogradation during freeze–thaw cycles. Furthermore, low value implies that the infant diet can be stored at refrigeration temperature for later use with less tendency to retrogradation or syneresis.

Pasting time is a measure of the cooling time. Sample A and B had significantly low values, while blended samples had significantly high values. Inclusion of treated bambara nut in the blend could be thought to increase the pasting time of the blend. The value recorded here favorably compared to 5.94–6.37 min for extruded African breadfruit snack (James & Nwabueze, 2014).

Pasting temperature provides an indication of the minimum temperature required to cook a given flour. High pasting temperature indicates high water‐binding capacity, gelatinization tendency, and lower swelling property of starch‐based flour due to high degree of association between starch granules. Samples in this study were not significantly different. The value (72.58–74.28°C) agreed with 73.1–75.2°C for cassava starch isolate (Ikegwu et al., 2009). The values were also in line with 67.74–75.82°C for complementary extrudates reported in a previous study (James & Nwabueze, 2014).

### 3.3 The sensory properties of germinated and fermented millet and bambara groundnut blend

The sensory attributes (Table 5) determined in this study showed marked variability among various samples except in aroma, taste, and appearance. The mean value for aroma ranged between 7.20 and 6.30. The result agrees with (Helen & Ragina, 2013) who reported 7.70–5.57 for maize‐based weaning food. However, the texture of the samples (7.30–5.75) was high compared to 5.90–4.05 for maize‐based weaning food (Helen & Ragina, 2013). The taste scores (7.45–6.35) of the samples were in line with the result (8.4–6.2) recorded for sweet potato and soya bean flour complementary food (Haque, Hosian, Khatun, Alam, & Gani, 2013). Meanwhile, the value recorded for appearance (7.25–6.45) was in compatibility with that of low‐cost complementary food (7.30) (Sadana & Chandni, 2004). The overall acceptability obtained in this study ranged between 8.05 and 6.95. This result was similar to the result recorded for sweet potato and soya bean flour complementary food (8.3–6.3) (Haque et al., 2013). The general acceptability of 100% fermented millet (sample B) had the highest acceptability, while sample C had the lowest rating.

**Table 5:** Sensory properties of the samples

Parameters	A	B	C	D	E	F
Aroma	7.20 ± 0.25	7.05 ± 0.30	6.30 ± 0.31	6.70 ± 0.21	7.05 ± 0.24	6.35 ± 0.35
Texture	6.50^abc^ ± 0.25	7.30^a^ ± 0.26	5.75^c^ ± 0.34	7.25^a^ ± 0.23	6.73^ab^ ± 0.24	6.15^bc^ ± 0.37
Taste	6.80 ± 0.31	7.45 ± 0.24	6.75 ± 0.40	6.35 ± 0.25	7.00 ± 0.32	6.90 ± 0.26
Appearance	6.45 ± 0.26	7.25 ± 0.23	6.70 ± 0.37	7.10 ± 0.28	7.20 ± 0.23	6.55 ± 0.28
General acceptability	7.15^bc^ ± 0.18	8.05^a^ ± 0.15	6.95^c^ ± 0.22	7.60^ab^ ± 0.21	7.60^ab^ ± 0.20	7.30^bc^ ± 0.24

Mean ± *SD* of triple determinations. Values followed by different subscript on a row are significantly different from each other (*p* < .05). A = 100% sprouted millet; B = 100% fermented millet; C = 95% sprouted millet and 5% sprouted bambara nut; D = 95% fermented millet and 5% fermented bambara nut; E = 95% sprouted millet and 5% fermented bambara nut; F = 95% fermented millet and 5% sprouted bambara nut.

## 4 CONCLUSION

From the result obtained, it can be concluded that sample C (95% germinated millet and 5% germinated bambara nut) has the suitable functional properties in respect to infant formulation.

## CONFLICT OF INTEREST

None declared.
